# Understanding illness experiences of patients with primary sclerosing cholangitis: a qualitative analysis within the SOMA.LIV study

**DOI:** 10.1186/s12876-023-02645-2

**Published:** 2023-01-13

**Authors:** Caroline Loesken, Kerstin Maehder, Laura Buck, Johannes Hartl, Bernd Löwe, Christoph Schramm, Anne Toussaint

**Affiliations:** 1 Department of Psychosomatic Medicine and Psychotherapy, University Medical Center Hamburg-Eppendorf, Hamburg, Germany; 2 1st Department of Medicine, University Medical Center Hamburg-Eppendorf, Hamburg, Germany; 3 Martin Zeitz Center for Rare Diseases, University Medical Center Hamburg-Eppendorf, Hamburg, Germany; 4 Hamburg Center for Translational Immunology (HCTI), Hamburg, Germany

**Keywords:** Chronic illness, Rare disease, Cholestatic liver disease, Interviews, Thematic analysis, Patient experience, Impact of illness, Coping

## Abstract

**Background:**

Primary sclerosing cholangitis (PSC) is a rare cholestatic liver disease with a largely unpredictable course. Due to limited treatment options, individuals may for many years suffer from distressing symptoms and the emotional burden of an uncertain future. The need to shift from cure to care of PSC has spurred an interest into patients’ health-related quality of life. Qualitative research in this context remains scarce. Hence, this study aimed to enrich the clinical understanding about the lived experience of PSC through a qualitative approach.

**Methods:**

A total of 20 patients with PSC were recruited at a specialist centre for autoimmune liver disease in Germany and engaged in semi-structured telephone-based interviews between March and June 2022. Verbatim transcripts were interpreted using inductive thematic analysis.

**Results:**

An overarching concept of ‘a wave-like experience’ was formulated to illustrate the dual and shifting nature of living with PSC. Reflecting upon this central idea, three major themes were generated to address important aspects of participants’ illness experiences: ‘Invisible presence’ focused on perceptions of suffering from a seemingly hidden illness that periodically reveals itself through specific trigger events. ‘Embracing the threat’ captured the psycho-emotional response shift to this chronic disease from a predominantly negative to a coping-oriented pattern with regular setbacks. ‘Between control and constraints’ uncovered restrictions that PSC enforces onto patients’ lives and their desire for controllability.

**Conclusions:**

The present study provides an in-depth look at the fluctuating tensions arising from a life with PSC. Insights on perceived invisibility, disease-related triggers of emotional distress and the complexity behind self-management highlight opportunities for enhanced clinical support of this patient group.

## Introduction

The diagnosis of a chronic disease such as primary sclerosing cholangitis (PSC) and its management bring about unique challenges to the lives of patients. Chronic disease not only affects the physical body, but also interferes with mental and social functioning [1]. Thus, investigating the psychosocial impact of PSC on affected individuals is considered essential to ensure high-quality patient-centred care over the long term.

PSC is a rare liver disease characterised by inflammation and scarring of the bile ducts, resulting in their abnormal narrowing (strictures), impaired bile flow and eventually liver cirrhosis [2]. Its progression remains largely unpredictable and can lead to an end-stage liver disease requiring transplantation [3]. While some individuals may be asymptomatic at first, the course of disease is associated with debilitating symptoms such as itching (pruritus), fatigue, yellow discoloration of the eyes and skin (jaundice) or abdominal pain [4]. Recent estimates suggest a prevalence of up to 16.2 cases per every 100,000 persons [5]. PSC is diagnosed more commonly in men (65–70%) between 30 and 40 years, yet it may occur at any age [6]. Approximately 70% of patients have a concurrent inflammatory bowel disease (IBD) [7]. Possible complications include bacterial cholangitis, clinically relevant high grade bile duct strictures, vitamin deficiencies and the development of hepatobiliary and colorectal cancer [8]. There exists no effective treatment or definite cure for PSC yet, hence disease management currently focuses on symptom alleviation and addressing complications [9].

In light of limited treatment options and the absence of validated risk stratification tools, patients with PSC may for many years live with distressing symptoms and the emotional burden of an uncertain future [10]. The need to shift from cure to care as part of the clinical management has spurred an interest into health-related quality of life (HRQoL) in relation to PSC [11–15]. Interestingly, subjective and non-life-threatening symptoms, in particular pruritus, rather than objective disease severity itself, were detected as important determinants of inferior HRQoL [11, 14]. Besides, the presence of comorbid and symptomatic IBD was linked to significant reductions in patients’ working ability [15]. Greater levels of psychological distress and depression were also identified amongst individuals with PSC [11–13].

While a number of quantitative studies assessed the impact of PSC on patients’ quality of life, they fail to sufficiently uncover what shapes individual perceptions and specific coping behaviours over time. An understanding of patients’ underlying illness beliefs is, however, essential for the work of healthcare professionals because such ideas directly relate to a number of important clinical outcomes such as satisfaction with consultations, future healthcare use, self-management behaviours and quality of life [16].

Leventhal outlines in his Common-Sense Model of Self-regulation (CSM) that illness perceptions are comprised of cognitive and emotional representations, which form the basis and direct guidance for coping strategies and illness-specific behaviours [17]. Hence, a detailed understanding may help us to appreciate patients’ responses to illness in terms of their feelings, thoughts and behaviours, which in turn influence relevant clinical outcomes. In this respect, qualitative research methods can facilitate a holistic and contextualised exploration of the multiple factors and processes involved in the chronic illness experience and thus highlight opportunities for enhanced patient care [18].

To date, however, very few studies used qualitative approaches in the context of PSC. Cheung et al. incorporated a content analysis of questionnaires as part of a mixed-methods approach on HRQoL and discovered a significant psychological burden amongst patients with PSC due to existential anxiety and social isolation [13]. In addition, Ranieri et al. conducted focus group interviews which highlighted episodes of psychological distress in affected individuals and their family members from diagnosis to post-transplantation [19]. Aside from the few disease-specific qualitative analyses, the burden of PSC has been explored in studies on rare diseases, which led to the identification of shared problems in different diagnoses including psychological distress, constraints in professional, personal and daily lives, stigmatisation and others lacking understanding [20].

Considering the dearth of knowledge about the lived experience of PSC, this study aimed to enrich the clinical understanding of patients’ beliefs and associated behaviours which can ultimately improve both communication in medical consultations as well as overall health outcomes. Accordingly, the following research questions were examined through a qualitative approach:

1. *How do patients with PSC perceive their illness and associated symptoms?*
2. *How do patients with PSC cope with their illness and associated symptoms?*
3. *How do their illness perceptions and management strategies evolve over time?*

## Methods

### Qualitative framework

This interview study applied the method of reflexive thematic analysis (TA) [21]. The chosen approach offers the possibility of an inductively-oriented exploration to identify patterns of meaning across a dataset in relation to the research questions. It further allows for theoretical flexibility in the development of themes to locate patients’ experiences within wider medical and social discourses [21]. The focus of analysis was mostly semantic, but also going beyond at times to uncover latent ideas and assumptions behind explicitly expressed content [22].

Regarding ontological and epistemological underpinnings, a critical realist position was combined with a contextualist view. In this way, the researchers recognise an inherent subjectivity in the production of knowledge and consider findings as situational and dependent on the circumstances in which data were collected and analysed [23].

### Set-up of the study

The present study was conducted at baseline assessment of the DFG-funded SOMA.LIV research project—a prospective single-centred cohort study involving patients with both primary biliary cholangitis (PBC) and PSC (Grant Nos.: TO 908/3-1 and SCHR 781/7-1). It is part of the interdisciplinary research unit SOMACROSS (RU 5211) entitled 'Persistent SOMAtic Symptoms ACROSS Diseases: From Risk Factors to Modification' [24].

### Participants and sampling

Recruitment was carried out at the YAEL-Center for Autoimmune Liver Disease located at the University Medical Center Hamburg-Eppendorf (UKE) in Germany. The specialist centre provides care for approximately 500 patients with PSC each year.

Embedded in the SOMA.LIV prospective cohort study, participants for this qualitative analysis were selected via purposeful sampling based on predetermined criteria of the overall project (see Table 1) while simultaneously aiming for maximum variation in terms of age, sex and duration of PSC. Thus, diversity was ensured and findings not restricted to typical cases only.

**Table 1:** Participant selection criteria

Inclusion criteria	Exclusion criteria
• Clinical diagnosis of PSC according to generally accepted criteria [25] • Age ≥ 18 years • Sufficient oral and written German language proficiency • Informed consent	• Advanced cirrhosis (defined by Child Pugh A score ≥ 8) or decompensated liver disease • History or presence of other concomitant liver disease (especially autoimmune hepatitis or chronic viral hepatitis B or C) • Presence of clinically significant untreated intercurrent medical condition associated with fatigue (i.e. hypothyroidism, anaemia, fibromyalgia, rheumatoid arthritis, systemic lupus erythematosus, active IBD and manifest depression) • Serious illness requiring immediate intervention • Florid psychosis • Substance abuse disorder • Acute suicidality

Starting in March 2022, all feasible patients for SOMA.LIV were contacted via telephone about 2 weeks prior to their (bi-)annual appointment at the YAEL-Center. Patients who agreed to participate received written study information by mail and were given the opportunity to address questions via telephone or on site before providing written informed consent for all parts of the mixed-methods study at their study visit. To conduct the interviews, additional telephone appointments were scheduled.

Interviews from 20 patients with diverse characteristics (see Table 2) were regarded as appropriate to provide sufficient information power for the generation of shared themes in this qualitative setting [26].

**Table 2:** Participant characteristics

Participant characteristics	Number [Mean]
*Sex*	
Female	10
Male	10
Age (range)	21–68 [43]
Years since diagnosis (range)	0–31 [11]
*IBD comorbidity*	
Ulcerative colitis	11
Crohn’s disease	3
None	6
*Place of living*	
Hamburg	9
Other part of Germany	11

### Data collection and processing

A semi-structured interview guide was developed in collaboration with research teams from three SOMACROSS projects (SOMA.LIV, SOMA.CK und SOMA.SOC) and drew on experiences from a previous qualitative study of our department on the psychological burden of patients with PBC [27]. It employed elements from the Structured Clinical Interview for DSM-5 (SCID-5) for somatic symptom disorder to investigate cognitive, emotional and behavioural dimensions in regard to symptom representations [28], thus accommodating a particular interest of RU SOMACROSS. In addition, open questions about changes in illness perceptions, emotional responses and related coping strategies were incorporated. In this way, important facets of illness experiences according to Leventhal’s CSM could be enquired while allowing room for further exploration. The pre-developed guide was approved by the Patient Advisory Board of the YAEL-Center and refined by means of trial interviews.

All interviews took place via telephone on a one-on-one basis between March and June 2022. They were audio-recorded following informed consent and ranged from 16 to 51 min with a mean duration of 29 min. The interviewer was female (CL) with a background in human medicine and qualitative market research, but no previous involvement in the care or medical treatment of this patient group. The spoken content was transcribed verbatim while assuring pseudonymisation [29], and prepared for analysis using MAXQDA 2022 (VERBI Software, 2021).

### Data analysis

Interview data were interpreted according to the six phases of reflexive thematic analysis as outlined by Braun and Clarke: (1) familiarisation, (2) coding, (3) generating themes, (4) reviewing themes, (5) defining and naming themes, and (6) writing up [21]. The first phase of data familiarisation was achieved through the process of transcription followed by rigorous re-reading of all transcripts and note-taking of first impressions. Next, an initial set of codes was generated by paying equal attention to each interview. Through a recursive process, additional codes were created and existing ones refined. After that, codes with similar meaning were grouped into clusters of shared ideas, which aided the inductive development of initial themes. These were then reviewed by relating back to original interview extracts as well as the overall research questions. Regular discussions with fellow researchers (AT, KM, LB, CS, JH) formed an integral part of the entire analytical process, facilitating reflexivity and the refinement of internally coherent and distinct themes with clear definitions, unique names and illustrative quotes. Finally, the findings were organised and reported starting with the overarching concept. Individual themes followed in line with Leventhal’s CSM to address cognitive and emotional experiences as well as management strategies of patients living with PSC.

### Ethical considerations

The present study was approved by the Ethics Committee of the Medical Chamber Hamburg on 21/12/2020 (Processing-No.: 2020-10196-BO-ff). It complies with the World Medical Association Declaration of Helsinki [30]. General study information was shared in both oral and written format and all participants provided informed consent. Data protection was ensured in accordance with the European General Data Protection Regulation as implemented on May 25th, 2018 [31].

## Results

**Fig. 1:**
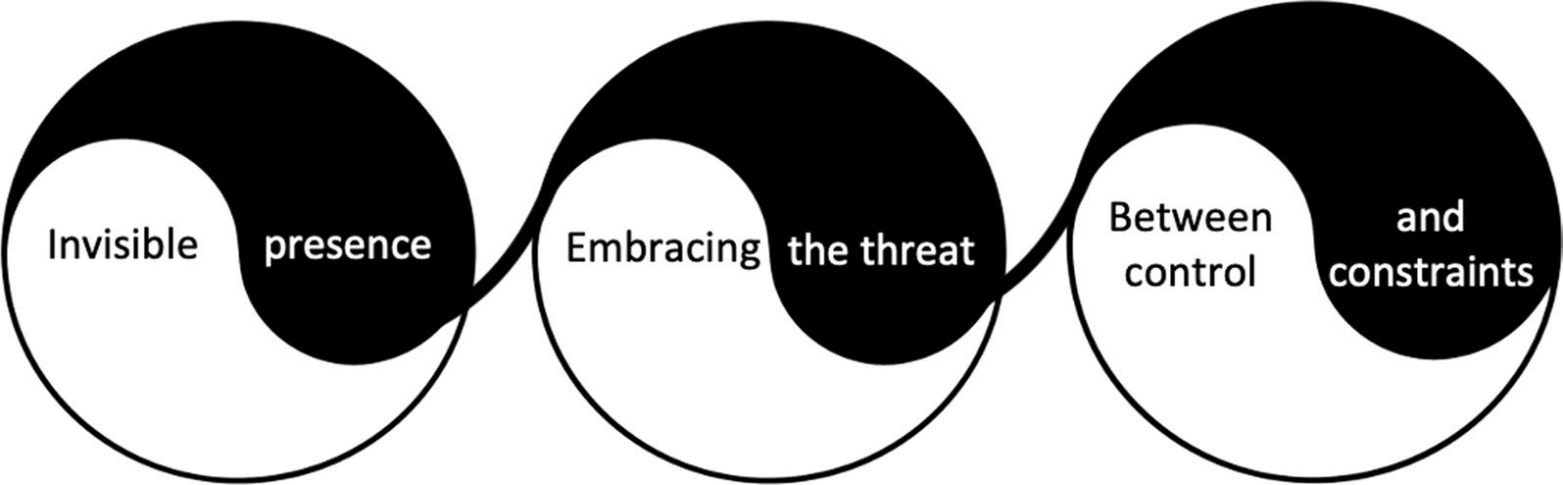
Three major themes composing ‘a wave-like experience’

The overarching concept of ‘a wave-like experience’ was generated to capture illness experiences of participants on a more abstract level. It illustrates the dual and shifting nature of living with PSC and is further reflected in the three major themes: ‘Invisible presence’, ‘Embracing the threat’ and ‘Between control and constraints’ (see Fig. 1). Each theme entailed opposing ideas concerning either cognitive perceptions, psycho-emotional responses or the question of controllability in relation to this chronic liver condition.

For the presentation of results in this manuscript, quotations were translated from German and partly edited for enhanced readability. Square brackets with context information were included where necessary. After each quotation, the participant ID is stated. P stands for participant and numbers represent individual positions in the interview process.

### Overarching concept: a wave-like experience

The overarching concept of ‘a wave-like experience’ is evident in all three themes. Participants described their illness-related thoughts, symptom burden and emotions as *“coming in waves”* (P3) or *“wave-like”* (P8).

The illness experience was depicted as *“a constant up and down”* (P1; P7) with good days when illness only played a minor role in daily life, when negative thoughts and associated fears moved to the back of the mind and a sense of good health and normality prevailed. On those days, participants felt in control over their health situation and the well-being aspect was paramount: *“Then I feel really good and also have lots of energy”* (P12).

On other days, however, when a wave of negativity overcame participants, for example, in form of distressing symptoms or disease complications, possibly paired with stressful life events, the threat of illness suddenly became apparent. In such situations, their PSC dominated the thinking process and constraints appeared uncontrollable, giving rise to negative emotions and a more pessimistic outlook: *“Maybe once every six months […] it overwhelms me to such an extent that I break down and cry because of it”* (P6).

As a result of this shifting nature, narratives may differ depending on personal circumstances at the time: *“If we had conducted this interview a month ago, I would have responded differently in terms of my current condition”* (P17). In this sense, the concept of ‘a wave-like experience’ is regarded as central to the understanding of each of the following themes and the overall experience of living with PSC.

### Theme 1: invisible presence

This theme captures the dichotomous nature of illness perception participants expressed based on their cognitive and physical experiences of PSC. On the one hand, an impression of ‘invisibility’ was reported due to missing or unspecific symptoms and subsequent problems of causal attribution, which affected decisions on disclosure of their condition in the social context. On the other hand, certain triggers were named that regularly pull the ‘presence’ of PSC into awareness and make public exposure inevitable at times.

For almost all participants, the time of diagnosis—often a discovery by chance—marked their first real encounter with the disease of PSC. Since many participants reported a lack of physical symptoms at this stage, diagnosis often posed a cognitive conflict: *“I had the kind of impression, they want to tell me I am sick and […] there is something wrong with my liver. I actually felt perfectly well”* (P9). Though medically defined ‘ill’ based on biological parameters, the absence of noticeable signs, since *“your liver doesn’t hurt after all”* (P20), hindered participants from making sense of their situation. Especially those diagnosed at young age with no prior experiences of serious illness and taken-for-granted assumptions about a smoothly functioning body noted *“that’s why I can’t really understand it, that there is something supposed to be wrong now”* (P20). For those with existing physical signs, diagnosis marked a turning point in their perception of past symptom experiences and led to the correction of earlier illness attributions: *“So I had thought that was neurodermatitis […] until I received the diagnosis sometime a year later”* (P1).

Nonetheless, the unspecific nature of associated symptoms left most participants with uncertainty about a possible connection to PSC, which reinforced the sense of suffering from an invisible illness. In the case of fatigue, other life circumstances, such as the birth of a new child, aging, too much work or a lack of exercise were often regarded as equally causal for the lack of energy. One participant illustrated in this context: *“This might have been my disposition anyway. I simply am a morning person. It’s difficult for me in the evenings. I always feel tired relatively early”* (P14). Narratives about more obvious and traceable symptoms such as *“severe* [abdominal] *pain and also an increased frequency of stool”* (P20) in relation to a comorbid IBD further support the notion of invisibility in their experience of PSC. In fact, many participants reported to forget about their liver condition in everyday life situations: *“When I am out and about here with my children or at work and have a lot to do there, then there is no ‘itching, itching’ in my head all the time”* (P10).

Concurrently, numerous participants recognised a certain omnipresence of their PSC, which always sits *“in the back of the mind”* (P1). They view their illness as *“a constant companion” *(P13) that inevitably is taken into consideration because it *“keeps resonating”* (P19) during important decision-making processes. Moreover, participants identified various triggers that periodically shift their illness and related symptoms into the focus of attention. Particularly times around regular medical check-ups were frequently connected with an intensification of cognitive and, in some cases, even physical flare-ups of their PSC: *“Especially prior to upcoming appointments, the itching feels much stronger because of my thoughts”* (P10). Besides, stress in general or specifically distressing life events were often linked to increased symptom severity and an acute awareness of their illness. Then, *“it always becomes noticeable in the liver region”* (P15).

The dichotomy between the hiding and revealing of PSC is also reflected in its social representation. Participants explained that their condition mostly remains unseen when encountering others: *“I believe my group of friends […] they rather don’t notice. […] One usually meets during leisure time. That’s a good time, that is pleasant. It’s not that apparent then”* (P14). Partly because of this perceived invisibility, the majority decided to not deliberately disclose their liver disease to a wider circle of family and friends. Some noted the burden this knowledge would impose on others: *“Because they have plenty of problems themselves”* (P13). Many participants also stated the aim of protecting themselves by eliminating unnecessary reminders of their illness or preventing exposure to inappropriate comments or *“clever advices”* given by others who *“cannot judge it in the end”* (P2). In this context, participants depicted experiences when their symptoms were simply dismissed as *„signs of ageing”* (P3) or played down by saying *“don’t stress yourself about it. There is nothing there”* (P10). Perhaps as a result of such incidents and to maintain the integrity of their self, participants mostly try to represent a normal and healthy image, thus actively contribute to the hiding of their illness: *“Whenever I meet someone or introduce myself, I don’t tell anything* [about my PSC]. *They ought to see me as a completely normal, let’s say a healthy person”* (P13).

Despite such efforts, some occasions appear to make disclosure inevitable. Participants indicated that their unusually high frequency of medical appointments or chosen abstinence from alcohol often attract attention and require revelation and explanation of their condition.

### Theme 2: embracing the threat

The second theme encompasses the psycho-emotional pattern participants show in response to their illness. It explores variations in emotional expressions and psychological adjustment depending on the stage of their illness experience, disease status and personal circumstances. Responses in this context ranged from shock, low mood, anger and anxiety to acceptance, hope, optimism and a sense of humour.

Feelings of shock were most often mentioned as initial response to a diagnosis of PSC. For many the news came unexpected. Participants felt overwhelmed and expressed concerns about the prospect of a shortened life-expectancy, which was particularly evident in those carrying responsibility for children: *“Since I am also a father now and still have relatively small kids, I was utterly shocked of course”* (P19).

Sadness, anger and a certain hopelessness were presented as key emotions following diagnosis, though expressions of sentiment were often toned down in the narratives. One participant said he *“was close to tears”* (P19), while others explained that it *“certainly does drag you down a little”* (P8) or acknowledged *“a kind of despair”* (P6). Some participants also described symptoms of depression due to the *“devastating or gloomy”* prognosis (P14). At this low point of their illness experience defined by mostly negative emotions and intense psychological strain, participants depicted their illness as a kind of *“weaker self”* (P17) or enemy that threatened feelings of normality.

Though to a lesser extent, the notion that symptoms ought to be overcome to pursue an ordinary life seemed persistent throughout. Associated feelings were described as *“gritting your teeth and getting through it somehow”* (P3). At the same time, participants repeatedly portrayed a sense of distress and anxiety of not meeting obligations due to their illness and burdening symptoms. Underlying feelings of guilt or fear to disappoint others appeared to play a major role in this context: *“There is simply this fear of not being able to manage the workload […]. To also let the team down in a way”* (P6).

Yet, over the course of time most participants found ways to incorporate their illness into their personal lives, which aided appeasement of the described inner turmoil to some degree. Symptoms were accepted as part of their PSC and participants managed to tolerate their existence because one *“knows the cause”* (P9) now and *“mentally fight*[ing] *against the pain […] makes the situation even worse”* (P3).

Following a period of adjustment, which was marked by a process of learning about the disease and comprehending one’s own physical reactions towards it, participants conveyed a rather optimistic and appreciative attitude towards their current health status. One participant explained that *“over the four years with this disease you somehow develop more of a feeling for what the body needs or whether you simply ought to rest […]. With that acceptance and understanding, I believe I can deal with it quite well”* (P8). By putting things into perspective, many began to see their illness and associated symptoms in a more positive light, making clear that *“others are doing far more poorly”* (P9) or *“by my standards, I am in a top condition”* (P18). In some cases, this newly developed optimism almost appeared unrealistic considering this uncurable and progressive disease: *“I also actually have confidence that it* [the PSC] *will possibly get better sometime. This is my own body after all”* (P19).

Nonetheless, subjective feelings of well-being and the overly optimistic outlook were closely linked to the momentary evaluation of the health threat. This connection became clear when participants began to report about the onset or intensification of burdensome symptoms and rising uncertainties about disease progression. In those cases, fear and anxiety dominated their narratives: *“You never know, for example, when they make an X-ray, whether on the next day they say ‘Oh, we detected something there.’ […] the fear is of course stronger, much stronger then”* (P14). It is, above all, this unpredictability regarding the course of their PSC that causes unease and underlying concerns, meaning that *“every now and then the sword of Damocles of a transplantation hovers over you"* (P15).

Perhaps to protect themselves against emotional distress or future disappointments, participants expressed the attitude that life and good health should be enjoyed in full here and now and one must *“not plan extremely far ahead”* (P13). A few participants even took it with humour when referring to their personal fate in relation to chances of longevity with PSC: *“I assume that I'm more likely to get run over by a bus”* (P18). With a certain acceptance of fate, participants seemed to keep a kind of emotional equilibrium.

### Theme 3: between control and constraints

The third theme explores the question of ‘who is in control?’ and how perceptions of imposed constraints versus feelings of controllability can influence patients’ choices of self-management strategies. It furthermore aims to illustrate what control may mean in the context of PSC—an incurable disease with an unpredictable course.

Participants affected by deteriorating physical symptoms described various constraints to both their personal and working lives. Some felt restricted due to a decline in physical performance, while others identified the impact on their cognitive abilities as particularly troublesome. One participant with regular episodes of nausea and pain explained: *“I used to also go hiking a lot […]. And now I just can’t be on my feet half a day anymore”* (P3). As a result of his more advanced liver condition, another participant suffering from cognitive deficits deplored that *“I make mistakes, which didn’t happen to me in the past”* (P15). Fatigue can equally control daily life to the extent that *“you don’t really have a choice but […] to get some rest”* (P14). At the same time, those affected by itching stressed the kind of mental control this symptom imposes on them, since it *“really drives you crazy”* (P20). Feelings of constraints, however, not only encompass executive functioning. Some participants noted how the illness limits their quality of life because it forced them to give up hobbies, reduce social activities or simply took away their sense of *“ease and joy”* (P10).

Despite restrictions, participants frequently expressed a desire to *“control the illness”* (P9). In fact, all of them reported a range of strategies with the primary objective to influence their health status in a positive way. Though knowing that a healthier diet, an abstinence from alcohol or increased activity levels would not lead to the healing of their liver condition, the employment of lifestyle changes strengthened their sense of agency and perceptions of active control over their current situation. In this regard, one participant described: *“I do yoga. That helps me a lot because it keeps the body flexible and it also makes me feel fitter”* (P12).

In addition, most participants developed individual self-care practices based on their own experiences to aid symptom management. One explained that *“the more dehydrated I am, the more prone to itching I get, so I discovered for myself that it is important to keep drinking plenty of water”* (P1).

As part of destressing their lives, several participants described how they re-evaluated priorities and lifetime goals to create a more manageable environment despite restricting symptoms, such as fatigue: *“I’ve now reduced my job to a simple job […] without anything else on top […] and I know that with what I have left now in terms of performance capacity, I will be able to maintain it”* (P17). Besides, some measures also implied a degree of cognitive control. Participants illustrated how distracting themselves helps to shift their focus away from disease-related thoughts or symptoms towards other aspects in life: *“I […] do sports, go out into fresh air, meet other people to get out of the situation and, above all, away from this scratching and my swirl of thoughts”* (P10).

For some, the process of acquiring appropriate self-management strategies required professional assistance. One participant acknowledged that she *“got help from a psychotherapist at the very beginning following diagnosis”* (P10).

When it came to the question of disease management as such, all interviewees clearly admitted personal limitations to control their unpredictable liver condition. Placing this aspect of management into the hands of a specialist doctor, who *“has results”* and *“sufficiently answers all the questions”* (P19), appeared to be the best choice. Participants recounted that with the aid of medicine, *“it* [i.e. the itching] *got better relatively quickly“* (P1). Such positive experiences in terms of effective symptom alleviation through medical intervention seem to have evoked a sense of controllability amongst participants. Indeed, they conveyed high amounts of trust in and reliance on medicine in relation to their PSC and associated complications.

One way to actively partake in the process of medical control was seen in the attendance of check-ups, meaning *“ultrasound, Fibro*[scan]*, MRI, and colonoscopy every six months or once every year”* (P17). Participants explained to *“give the best and regularly go to the examinations”* (P9) with the justification that it is *“because of the risk, to be close to it* [i.e. cancers]*”* (P8). A general awareness, that early detection of disease complications may be beneficial for their future prognosis, perhaps explains high adherence levels to this rigorous medical surveillance scheme amongst this group of patients. Besides, regular attendance possibly creates a sense of exerting at least partial control over the course of their illness.

Nevertheless, the effort made to master their PSC comes with yet other constraints. For many participants the time-consuming medical appointments require extensive planning. Some drive many hours to the specialist centre. For such reasons, one participant expressed his frustration about having to *“sacrifice two days of vacation for these examinations”* (P17), while another with more than 30 years of illness experience reveals that *“after all, it comes more and more into awareness that all of that feels just a bit exhausting”* (P9).

## Discussion

The main objective of this qualitative interview study was to gain deeper insights into illness experiences of PSC. Using inductive thematic analysis, the overarching concept of ‘a wave-like experience’ was developed to reflect the dichotomous and shifting nature of the narratives on illness experiences on the whole. As part of this central idea, three individual themes were generated to portray aspects of major importance to this patient group:

1. ‘Invisible presence’ focused on perceptions of suffering from an illness that seems invisible both to the self and social environment, while at times clearly revealing its presence due to internal as well as external trigger events.
2. ‘Embracing the threat’ shed light on psycho-emotional responses to a life with PSC, which were characterised by a shift from a predominantly negative to a more balanced and coping-oriented pattern with periodic setbacks over the course of time.
3. ‘Between control and constraints’ uncovered the complexity behind the management of this incurable disease with a largely unpredictable course and the various imposed restrictions that hamper controllability.

### Findings in relation to existing literature

In the following section, the overarching concept will first be placed into context while individual themes are discussed according to key elements of Leventhal’s CSM, namely cognitive representations, emotional responses as well as aspects of self-management and control.

The concept of ‘a wave-like experience’ clearly resonates with Paterson et al.’s model of shifting perspectives between ‘illness’ and ‘wellness’ as part of the chronic illness experience [32]. Participants’ narratives indicate drifts between a perceived invisibility of their PSC when oriented towards health and a positive mindset in daily life versus a clearly felt presence in physical, cognitive or emotional terms as a results of illness-related trigger events. According to the model, each perspective holds specific functions at the time. While illness-in-the-foreground may be protective and help a person to learn about the disease and come to terms with it, wellness-in-the-foreground can be regarded as an opportunity for meaningful changes in self-identity and a focus away from the primarily diseased body towards other aspects of life [32].

With that in mind, more traditional assumptions that those facing a life-threatening diagnosis follow along a linear pathway from denial towards acceptance, as suggested by the Kübler-Ross model [33], may not reflect the complexity and fluctuating tensions of living with PSC. The current study surely confirms that acceptance forms an integral part of the adjustment process, but also illuminates continuing attempts to direct the focus away from illness, with the aim of moving on to a more normal life over the long-term. In this sense, findings accord with the ‘quest for ordinariness’ that Kralik describes as a transition towards incorporating chronic illness into everyday life and regaining a sense of balance and control, which was relinquished in the ‘extraordinary' phase of turmoil and distress following diagnosis [34]. For many participants this transition required considerable time and, in some cases, also professional assistance.

In terms of cognitive representations, the perceived invisibility of PSC and its association with social disclosure forms a major theme amongst participants. In fact, challenges in regard to illness attribution have been recognised in other chronic conditions with invisible signs such as osteoporosis [35], end-stage kidney disease [36] or systemic lupus erythematosus [37]. Invisibility leaves decisions of disclosure up to the patient. In this study, participants expressed their preference for not revealing their PSC to a wider circle of family and friends. This inclination to hide the disease due to a generally healthy appearance can equally be found in patients with ulcerative colitis (UC) or rheumatoid arthritis (RA) [38, 39]. Adding to this, research on the chronic and in parts invisible condition of scleroderma suggests that the rarity of the illness adds to the lack of understanding by others, thus disclosure not only means to deal with people’s reactions but results in an extra burden of having to explain the disease [40]. Such findings strongly resonate with participants’ experiences in this study.

Considering emotional facets of living with PSC, the time following diagnosis was characterised by shock, anger, sadness and, for some, a period of immense psychological strain. These initial reactions echo Ranieri et al.’s findings on psychological distress in patients with PSC and are comparable to those seen in people facing life with incurable cancer [19, 41]. The present study further illuminates that hope, optimism and a sense of humour seem to help participants in dealing with their illness over time. In fact, hope was linked to a better HRQoL in patients with Psoriasis, also an incurable chronic disease [42]. In addition, hope and optimism were both found to negatively correlate with anxiety and depression levels amongst patients with cancer [43]. Besides, Fournier et al. confirmed the positive long-term effect of optimistic beliefs on the adaption to different chronic diseases, but also informed that perceived controllability may lead to distress when patients were confronted with an uncontrollable illness [44].

The aspect of self-management nonetheless proved invaluable for participants to maintain a sense of control over their general well-being on a daily basis. Disease management as such, however, meant complete reliance on specialist doctors. With respect to the health locus of control theory (HLOC) [45], literature on chronic illness mostly emphasises the positive effects of holding an internal HLOC, meaning a belief that health lies in one’s own control, as opposed to an external one, where control is placed into the hands of others or chance [46, 47]. When facing a seemingly uncontrollable disease like PSC, things might indeed be more complicated. Findings by Burish et al. from their work with cancer patients imply that in medical situations where little personal control is possible, an external HLOC may in fact be advantageous for its positive impact on psychological arousal and affect [48]. Adding to this, Affleck et al. found that attributions of personal control proved helpful for daily symptom expression in patients with RA, but that those who relinquished control to their doctors for the overall course of their illness showed less mood disturbances and more positive psychosocial adjustment [49]. To ensure this selective control strategy in the context of PSC, patients need to have regular access to trusted specialists in the field. Otherwise, they are forced to rely on self-management and, as Ranieri et al. illuminates, psychological distress may arise [19].

### Strengths and limitations

This qualitative study comprehensively investigates illness experiences of patients with PSC. It thereby adds a novel dimension to the understanding of patient responses to this chronic liver condition by stressing the shifting nature in well-being instead of primarily building on a chronological narrative towards adjustment [19].

Telephone-based interviewing ensured a familiar and private environment, wherefrom participants could talk about sensitive issues. Previous research has shown its validity in the administration of structured clinical interviews, while high rates of acceptability were found amongst patients with a life-threatening disease [50, 51]. Although the lack of facial expressions and body language may arguably deprive the communication process from essential cues, findings suggest that a removal of visual distractions by using a telephone not only eliminates potential biases based on appearance and behaviour, but also places greater focus on the spoken word and the importance of clear articulation [52]. Thus, the overall quality of verbal communication may be improved, resulting in rich data for the purpose of this thematic analysis.

The selection of participants was limited to one centre of expertise in a metropolitan area of Germany, which possibly affected patients’ experiences with the medical system and excluded those who might not receive regular medical attention or adequate treatment. Exclusion criteria (see Table 1) were chosen in line with the overall aims of the SOMA.LIV project, however, the elimination of various comorbidities reduced patient diversity to some extent and restrained from deeper investigations as part of this qualitative approach. Besides, participation was based on voluntariness, meaning that illness experiences of those who avoid reflecting upon them remain unknown.

Finally, this explorative research did not cover patient experiences at later disease-stages, i.e. immediately pre- or post-transplantation, because of the distinct emotional burden and special needs that arise from this intervention. Future research would need to address this area to close the gap in knowledge.

### Clinical implications

The complex nature of living with PSC suggests for a more prominent role that healthcare professionals could play in supporting this patient group besides regular medical examinations. Since not every physician may be familiar with this condition, the European Reference Network on Hepatological Diseases (ERN RARE-LIVER), for example, offers qualified information and ways of collaboration for healthcare professionals, and patients equally, to foster a deeper understanding of rare liver diseases like PSC and improve communication in patient management.

Nevertheless, as patients’ experiences and their ability to cope with this illness seem to shift and, especially prior to upcoming examinations, fears and worries appear to increase, these aspects should always be asked about and re-evaluated as part of medical consultations. Meanwhile, the desire for self-management and personal control expressed by the patients may ask from healthcare professionals to additionally act as mediators by referring to local patient organisations. These organisations not only offer disease-specific information or hold regular events to improve health literacy amongst patients, they can also help individuals searching for self-support groups.

Finally, the development of evidence-based tailored interventions for patients with rare liver conditions marks a long-term aim of the SOMA.LIV project. Findings from this qualitative study will complement the available quantitative data and can thus be seen as one important step towards a better understanding of the experiences and needs of these patients.

To conclude from this analysis, the following general implications for clinical practice and patient management could be summarised:

- Healthcare professionals should acknowledge existing shifts between different illness perspectives in patients with PSC and be aware of tensions arising especially before upcoming medical appointments.
- Newly diagnosed patients should be educated that their own well-being may return following a time of adjustment to the changed living conditions.
- In this respect, healthcare professionals must be sufficiently sensitive to detect patients who struggle with the integration of PSC into their personal life and offer psychological support early.
- In addition, healthcare professionals should support the search for individual self-care practices and encourage patients to actively participate in the disease management process because it helps them to feel at least partially in control over their health situation.
- After all, greater awareness must be raised about this kind of rare and chronic liver condition with invisible signs amongst non-specialist healthcare providers and the public in general. Sensitisation may not only ensure early referral to a specialist centre but moreover reduce the perceived burden of illness disclosure in different socio-cultural settings.

## Conclusions

This qualitative interview study uncovered themes of major importance to patients with PCS as part of their illness experiences in everyday life. It shed light on a perceived invisibility of their PSC in both the personal and social context, highlighted disease-related triggers of emotional distress and captured attempts of self-management and control despite restricting symptoms and limited treatment options. In addition, a dual and shifting nature in illness perceptions, psycho-emotional responses and coping behaviours was identified as a key to understanding the lived experience of PSC.

The findings not only produce important implications for the clinical management of this patient group but may also help to raise awareness about the complexity behind a life with this rare and chronic liver condition.

## Data Availability

The data that support the findings of this study are available upon reasonable request from the corresponding author, CL. The data cannot be made publicly available due to their containing information that could compromise the privacy of research participants.

## Abbreviations

CSM: Common-Sense Model of Self-regulation
DFG: Deutsche Forschungsgemeinschaft, German Research Foundation
DSM-5: Diagnostic and Statistical Manual of Mental Disorders, Fifth Edition
HLOC: Health locus of control
HRQoL: Health-related quality of life
IBD: Inflammatory bowel disease
PBC: Primary biliary cholangitis
PSC: Primary sclerosing cholangitis
RA: Rheumatoid arthritis
RU: Research unit
TA: Thematic analysis
UC: Ulcerative colitis
